# Total Knee Arthroplasty with a Ti6Al4V/PEEK Prosthesis on an Osteoarthritis Rat Model: Behavioral and Neurophysiological Analysis

**DOI:** 10.1038/s41598-020-62146-0

**Published:** 2020-03-24

**Authors:** Mathieu Lecocq, Jean-Marc Linares, Julien Chaves-Jacob, Thelma Coyle, Sandrine Roffino, Marielle Eyraud, Didier Gigmes, Patrick Decherchi, Erick Dousset

**Affiliations:** 1 Aix-Marseille Université, CNRS, Institut des Sciences du Mouvement: Etienne-Jules MAREY (UMR 7287), Equipe « Plasticité des Systèmes Nerveux et Musculaire » (PSNM), Parc Scientifique et Technologique de Marseille-Luminy, CC910, avenue de Luminy, F-13288 Marseille, Cedex 09 France; 2 Aix-Marseille Université, CNRS, Institut des Sciences du Mouvement: Etienne-Jules MAREY (UMR 7287), Equipe « Conception Bio-Inspirée » (CBI), Parc Scientifique et Technologique de Marseille-Luminy, CC910, avenue de Luminy, F-13288 Marseille, Cedex 09 France; 3 Aix-Marseille Université, CNRS, Institut des Sciences du Mouvement: Etienne-Jules MAREY (UMR 7287), Equipe « Groupe Interdisciplinaire en Biomécanique Ostéoarticulaire et Cardiovasculaire » (GIBOC), Parc Scientifique et Technologique de Marseille-Luminy, CC910, avenue de Luminy, F-13288 Marseille, Cedex 09 France; 4 Aix-Marseille Université, CNRS, Matériaux Divisés, Interfaces, Réactivité, Electrochimie (UMR 7246), Equipe « Electrochimie des Matériaux » (ElMa), Faculté des Sciences et Techniques de Saint-Jérôme, 141, Traverse Charles Susini, F-13013 Marseille, France; 5 Aix-Marseille Université, CNRS, Institut de Chimie Radicalaire (UMR 7273), Equipe « Chimie Radicalaire Organique et Polymères de Spécialité » (CROPS), Faculté des Sciences de Saint-Jérôme, CC542 - Avenue Escadrille Normandie Niemen, F-13397 Marseille, Cedex 20 France

**Keywords:** Bone, Neurophysiology

## Abstract

Arthroplasty is a surgical procedure to restore the function of the joint of patient suffering from knee osteoarthritis. However, postoperative functional deficits are reported even after a rehabilitation program. In order to determine the origin of functional deficits of patient suffering from knee osteoarthritis and total knee arthroplasty, we developed a rodent model including a chemically-induced-osteoarthritis and designed a knee prosthesis (Ti6Al4V/PEEK) biomechanically and anatomically adapted to rat knee joint. Dynamic Weight-Bearing, gait kinematics, H-reflex from *vastus medialis* muscle and activities from metabosensitive III and IV afferent fibers in femoral nerve were assessed at 1 and 3 months post-surgery. Results indicate that knee osteoarthritis altered considerably the responses of afferent fibers to their known activators (i.e., lactic acid and potassium chloride) and consequently their ability to modulate the spinal sensorimotor loop, although, paradoxically, motor deficits seemed relatively light. On the contrary, results indicate that, after the total knee arthroplasty, the afferent responses and the sensorimotor function were slightly altered but that motor deficits were more severe. We conclude that neural changes attested by the recovery of the metabosensitive afferent activity and the sensorimotor loop were induced when a total knee replacement was performed and that these changes may disrupt or delay the locomotor recovery.

## Introduction

Osteoarthritis (OA) is a common degenerative joint disease, characterized by a failure of cartilage self-repair. It originates from a stress occurring on the synovial joint tissues including joint cartilage, subchondral bone, ligaments, menisci, periarticular muscles, peripheral nerves or synovium and leads to inflammatory reaction^1–3^. Mainly, OA involves hands and weight bearing joints, especially the knee which appears to be the most affected^4^. The main symptoms of knee OA are pain and motor limitations related to joint deformity and weakness of surrounding muscles^1,5,6^. When these symptoms become unsustainable, patients often resort to total knee arthroplasty (TKA) also called total knee replacement which is the gold standard treatment for end-stage knee OA. The main goals of a TKA are to restore knee function and alleviate pain by correcting knee joint deformity. This process has steadily increased over the past decades and is projected to grow drastically in the coming years in line with prevalence of obesity, non adapted physical activities and osteoarthritis^7,8^. Establishment of a knee prosthesis is a major procedure because it results of an inevitable joint capsule opening, menisci removal and cruciate ligaments disruption causing important neuromuscular disturbances^9^. Postoperative functional deficits are mainly characterized by proprioception and kinesthesia impairment, strength loss, voluntary activation deficit and atrophy of both quadriceps and hamstring muscles^9–12^. To reduce these functional deficits, physical therapies, mainly based on neuromuscular electrical stimulation (NMES) and voluntary physical activities, are commonly recommended. Unfortunately, despite these strategies, patient recovery remains often partial as demonstrated by several case reports^11–14^. For example, it was concluded that even 6 to 13 years post-surgery and despite physical rehabilitation, quadriceps muscle weakness persisted in people with a TKA compared with healthy people^15^. Currently, no universal rehabilitation protocol for patients who have undergone a TKA exists^14^. Moreover, the contralateral limb can also exhibit functional deficits even in the absence of joint disease, because of the functional limitation imposed by the operated knee^10^. Indeed, several studies demonstrated that quadriceps/hamstring muscles weakness and associated co-activation occurred likewise bilaterally resulting in knee stiffness^16,17^. Thus, ability to use appropriate motor strategies decreases and related gait functionality are altered because to a switch to slower and safer patterns^9,18–20^. Over time, this phenomenon could lead to contralateral knee degradation which cannot sustain higher repeated weight bearing strain, and could finally result in a second TKA process of the contralateral healthy knee. This adaptive strategy is presumed to increase joint stability during prolonged activity^17^ and reflects the role of sensorimotor loop in overcoming task constraints despite heavy neuromuscular deficits. However, during the first year after surgery, Swinkels *et al*., reported a 24.2% of postoperative fall rate for patients who have had a TKA and a 45.8% fall rate for patients identified as fallers prior to surgery showing that nervous compensation strategies are not sufficient to ensure the safety of operated patients^21^. The underlying mechanisms of neuromuscular changes in patients undergoing a TKA are not well understood^9,22^. However, in OA and TKA context, joint and muscular lesions induce afferent impairment leading to central nervous changes. Indeed, sensorimotor loop and central motor drive are modulated by chemical and mechanical changes in skin, joints and muscles^23–25^. More precisely, motor control is partially regulated by both groups I/II (mechanosensitive) and groups III/IV (mechano- and metabosensitive) joint and muscle afferents. As explained above, in context of OA and/or TKA, joint, cutaneous, and mainly muscle mechanosensitive (proprioceptive) information from group I and II could be altered. However, afferent fibers from groups III and IV could play a leading role because they are activated by different chemical (mainly potassium, lactic acid, inflammatory mediators) and mechanical stimuli (intramuscular pressure)^26^ and because their activation leads to modulation of reflexes mediated by group I and II afferent fibers, α-motoneuron activity and descending motor drive^24,25^. Thus, any change in III and IV afferent activity may delay the recovery because of an inappropriate neural strategy of recovery.

Furthermore, as suggested in the literature, it seems relevant to explore the neuromuscular adaptations from patient suffering from knee osteoarthritis and TKA in order to more efficiently target the origin of functional deficits and improve long-term outcomes^9,22^.

In order to determine the origin of the functional deficits observed after knee osteoarthritis and TKA, we developed a rodent model including a chemically-induced-OA and a knee prosthesis biomechanically and anatomically adapted to rat knee joint. To the best of our knowledge, only two studies have already attempted to create and to implant a knee prosthesis on rat^27,28^. However, both of these models were not really suitable as their shape did not respect knee joint surface, leading to joint instability and bone fractures. In this study, the first challenge was, after modeling the knee joint, to design a total knee prosthesis complying normal anatomical and biomechanical features of the rat knee joint. Then, the prosthesis was implanted after a chemically-induced-OA and Dynamic Weight-Bearing, gait kinematic, H-reflex from *vastus medialis* muscle and activities from III and IV afferent fiber in femoral nerve were assessed at 1 and 3 months later. The study was completed by a histological analysis of the joint.

## Materials and Methods

### Animals and experimental design

Forty-eight adult male Sprague Dawley rats (12 weeks old), weighing 400 g (Centre d’Elevage Roger JANVIER^®^, Le Genest Saint Isle, France), were housed in plastic cages at 22 °C with a 12 h light/dark cycle. Food (Safe^®^, Augy, France) and water were available *ad libitum*. An acclimation period of one week was allowed before the initiation of the experiment. All animals were weighed before each experimental step.

Rats were randomly assigned to four experimental groups: 1) Control group (n = 12) which received no treatment; 2) Sham group (n = 12) in which TKA surgery approach was made without injuring knee joint capsule; 3) MIA group (n = 12) which received knee intra-joint injection of mono-iodoacetate leading to an OA and 4) TKA group (n = 12) undergoing a TKA three weeks after a mono-iodoacetate injection. This last group remains consistent with clinical reasons (i.e., OA) leading to TKA prescription.

Each of these four groups was divided into two subcategories according to the recovery time before the electrophysiological analysis (i.e. one month - M1 or three months - M3).

In order to design the total knee prosthesis, three additional rats were sacrificed prior to the study. Their left tibial and femoral bones were collected and modeled.

### Ethical approval

Anesthesia and surgical procedures were performed according to the French law on animal care guidelines. The Animal Care Committees of *Aix-Marseille Université* (AMU) and *Centre National de la Recherche Scientifique* (CNRS) approved our protocols. Individuals conducting the research were listed in the authorized personnel section of the animal research protocol or added to a previously approved protocol (License A 13 01306). Furthermore, experiments were performed following the recommendations provided in the Guide for Care and Use of Laboratory Animals (U.S. Department of Health and Human Services, National Institutes of Health) and in accordance with the European Community’s council directive of 24 November 1986 (86/609/EEC), the ARRIVE Guidelines and the U.K Animal (Scientific Procedure) Act,. 1986. All these guidelines were carefully followed. No clinical sign of pain or unpleasant sensations (i.e. screech, prostration, hyperactivity, anorexia) or paw-eating behavior was observed throughout the study.

### Prosthesis design

**Figure 1:**
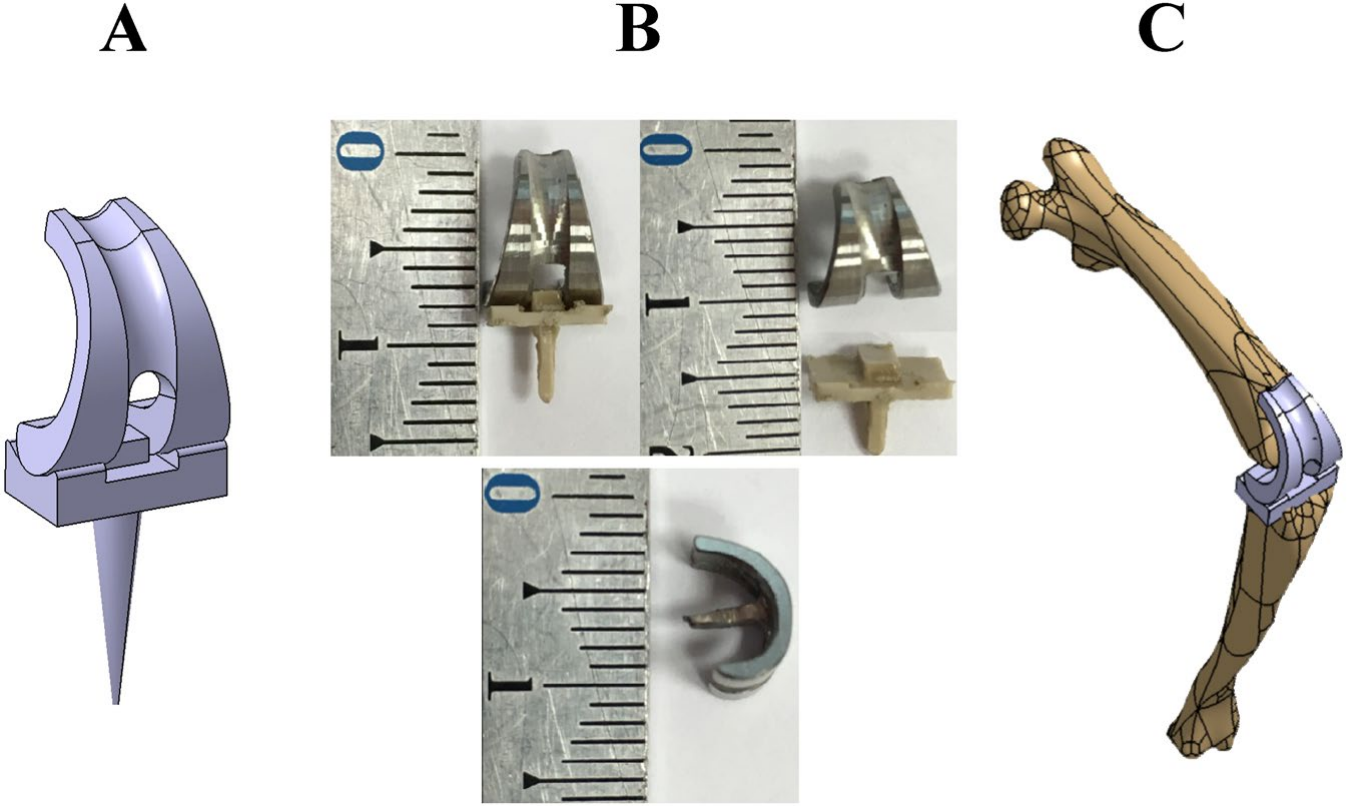
*Total knee prosthesis*. **(A)** Rat tibial and femoral bones are three-dimensionally modeled and components of the total knee prosthesis are designed. (**B**) Photographs of the total knee prosthesis: front (upper) and profile (lower) views. (**C**) Full design of the prosthesis positioned between the tibial and the femoral bones.

In order to create a prosthesis that is biomechanically and anatomically adapted to the rat knee joint, the same methods employed in human clinic were used. Briefly, collected tibial and femoral bones were immersed during 4 hours in boiling water with antibacterial soap in order to completely separate bones from surrounding tissue. Bones were digitalized using an optical measuring system (3D Scanner ATOS 3, GOM^®^, Braunschweig, Germany) and the data were treated by an inverse engineering methodology of a computer-assisted design system (CATIA V5, Dassault System^®^, Velizy, France). Thus, three-dimensional (3D) numerical model of rat knee joint was obtained. From this model, a computer-assisted design system allowed to design the geometrically-adapted tibial and femoral components of the future prosthesis **(**Fig. 1**)**. Femoral and tibial components were respectively machined from a titanium alloy (Ti6Al4V) and a polymer material (polyether-ether-ketone; PEEK) by a 5-axes micro-milling machine (US 20, DMG-Mori^®^, Leonberg, Germany). The programming process for machining each piece was performed by an ISO standard program generated by the CATIA V5 system.

### Osteoarthritis induction

Monoiodoacetate (Sigma Aldrich^®^, St. Louis, MO, USA) injection is recognized as an effective technique allowing histologic and morphologic changes in joint cartilage, closely similar to those observed for patients with OA^29–32^. By promoting glyceraldehyde-3-phosphate dehydrogenase activity inhibition of chondrocytes, mono-iodoacetate induces glycolysis disruption resulting finally in progressive chondrocytes cell death^32,33^. Thus, an injection of 1 mg of mono-iodoacetate diluted in 50 μl of saline is sufficient to induce maximal functional deficits by the 14 days post-injection^30^.

In order to induce knee osteoarthritis, rats from MIA and TKA groups were slightly anesthetized by a transcutaneous intramuscular injection of a mixture of 0.65 ml of ketamine (Ketamine 1000, Virbac^®^, Carros, France) and 0.25 ml of chlorpromazine (Largactil^®^, 0.1 ml per 100 g, Sanofi Aventis Laboratory^®^, Paris, France). Then, a single intra-joint injection of mono-iodoacetate (1 mg diluted in 50 μl of saline) was performed in the left knee joint with a microsyringe (Ito Corporation Exmire^®^, Shizuoka, Japan).

### Total knee arthroplasty surgery

Rats were anesthetized by an intraperitoneal injection of a mixture of medetomidine hydrochloride (0.5 mg.kg^−1^, Medetor^®^, Virbac^®^) and ketamine (75 mg.kg^−1^). The left hindpaw was shaved, and, according to the human parapatellar approach, a 1.5-cm skin incision was made along the medial border of the patellar tendon. The latter was laterally reclined and maintained in this position with retractors. In order to improve joint exposure, knee was positioned in flexion allowing thereby cruciate ligaments section and meniscectomy. Then, a portion of joint surface of the femoral condyles and tibial tray, with volume and shape equivalent to femoral and tibial component of the prosthesis, was removed using a micro milling/grinder machine (Dremel^®^ 300 series multitool, Robet Bosch^®^ SAS, Saint-Ouen, France). Suitable femoral and tibial guides were specially designed and used to ensure proper placement of tibial and femoral components. These guides were also used as drill barrels to introduce tibial and femoral prosthesis stems into bones to optimize implant-to-bone adhesion. As in orthopedic surgery, femoral and tibial components were fixed with bone acrylic cement (CEMFIX 1, Teknimen^®^, L’Union, France) spread on the inner surface on each component. The fixation was performed by manual pressure exerted for several seconds until cement dries to ensure good attachment. Following this drying delay, knee was positioned in extension to check the well alignment between femoral and tibial prosthesis. Then, patellar tendon was replaced above the knee, sutured to muscular edge and skin was subsequently sutured (Vicryl^®^ 3–0, Ethicon^®^, Johnson & Johnson^®^ Medical SAS, Issy-les-Moulineaux, France). Finally, a 0.2 ml injection of local anesthetic (Lidocaine T7394c, Sigma-Aldrich^®^) was performed subcutaneously around the implantation site to minimize post-operative pain.

### Behavioral tests

#### Dynamic weight-bearing

Weight distribution is considered as a relevant index of pain and is based on the assumption that nociceptive inputs can alter paw weight distribution^30,34,35^. Dynamic weight-bearing (DWB) device (Bioseb^®^ Development, Vitrolles, France) consists of a biometric floor instrumented cage (Captor surface: 10.89 mm^2^, Captor threshold: 0.1 g; Matrix Sensor 5250 type:/10, Tekscan^®^ Inc., Boston, MA, USA) allowing discrimination and measurement of the pressure exerted by each rat paw during short periods of free moving^34^. However, the paw weight-bearing can only be analyzed in static position periods during trials. Thereby, weight distribution functional deficits associated with trauma of central and/or peripheral nervous system were objectively identified^34–36^.

In the present study, each rat was placed on DWB device and pressures (g/unit area) of each paw were recorded during two trials of 5 min each. The first step of data processing was to assign each pressure zone to the corresponding rat paw by means of a synchronized video-recording and scaled map of the stimulated captors. Only stable paw pressures of at least 0.2 s were retained. Then, home-made MatLab^®^ functions allowed us to: i) normalize the time periods spent on 2 and 4 paws by the total time spent in static position for each recording, ii) normalize pressures in rearing condition (pressures exerted only on the two hindpaws) to the total weight of the animal and iii) normalize pressures in standard condition (pressures exerted by the four paws) to the total weight of the animal.

#### Gait kinematics

Two weeks before data acquisition, animals were trained within an enclosed plexigas^®^ walkway (L150 x W9 x H40 cm^3^) which was illuminated by two spotlights. A camera (iPhone 5, Apple^®^ Cupertino, CA, USA) was positioned perpendicularly to the walkway allowing the recording of rat locomotion. Prior to data acquisition, the left hindpaw of each rat was shaved and four anatomical landmarks were drawn with an indelible marker on the greater trochanter, knee lateral condyle, ankle medial malleolus and the 5^th^ metatarsal bone, thus identifying the hip-knee, knee-ankle and ankle-paw bone segments. Rats were successively placed on the right side of the walkway such that their left side was exposed to the camera lens and a sound stimulus was used to initiate their locomotion. Each rat accomplished five trials which were validated only if they performed at least four consecutive gait cycles at a steady pace in the field of view of the camera. Then, video analysis software (Kinovea^®^, Free software, Association Kinovea, Le Taillan Médoc, France) was used to extract the 2D coordinates of the four joint markers. The angular evolution of knee and ankle during gait cycles was then reconstructed. Based on 5^th^ metatarsal height, stance and swing phases of each gait cycle were identified. In order to generate average group profiles, both phases of all trials were normalized with respect to time. According to previous study, the transition from stance into swing phase was estimated to occur around 60% of gait cycle^37–39^.

### Electrophysiological recordings

Rats were anesthetized by an intraperitoneal injection of urethane (0.12 g/ml; 1 ml/100 g; Sigma-Aldrich^®^). The inner part of the healthy hindpaw was incised and femoral artery was isolated from surrounding tissues. In order to allow injections chemical known to activate III and IV afferent fibers, a catheter was inserted into the proximal portion of the artery toward the abdominal fork. The operated/treated hindpaw was also incised in order to isolate femoral nerve. Rat was then placed in a Faraday cage, to avoid signal noise, and femoral nerve was placed on stimulation electrodes (MLA 1204 needle electrodes, 29 gauge, 2 mm pin; ADInstruments Ltd., Paris, France) and covered with paraffin oil to prevent drying. To collect electromyographic activity (EMG), two electrodes were inserted into left *vastus medialis* muscle. A supplemental reference electrode was positioned on neutral tissue. Using a differential amplifier, the nerve signal was amplified (2 kHz) and band-passed filtered (100 Hz to 3 kHz).

#### H-reflex

In order to evaluate the maximal amplitude of the H-reflex, stimulation intensity was progressively incremented (by 0.01 mA) from motor threshold to obtain maximal amplitude of the H-reflex (H_max_). Since H_max_ was obtained, electrodes position remains unchanged. In order to control H_max_ stability, a series of twenty stimulations was then performed at a frequency of 0.1 Hz. After 10 minutes of rest, a new series of twenty stimulations was performed during which a mixture of 0.5 ml of potassium chloride (KCl, 10 mmol/l) and 0.5 ml of lactic acid (AL, 25 mmol/l) was injected through the catheter at the 6^th^ stimulation. This protocol allowed verifying the effect of III and IV muscle afferent activation on H-reflex response. Indeed, it was previously demonstrated that specific activation of these afferent groups induced a H-reflex inhibition^40^. Thus, fourteen post-injection reflexes were averaged and compared to the six pre-injection reference reflexes. Then, stimulation intensity was increased in order to attain the maximal amplitude of the M-wave (M_max_). The same injection protocol was performed to verify M_max_ stability and the absence of M_max_ response when III and IV muscle afferents were activated. So, to overcome the impact of changes in muscular conduction properties, results were expressed as a ratio of H_max_/M_max_. Amplitude variation occurring after chemical injection was only due to spinal and supra-spinal regulatory mechanisms.

#### III and IV afferent fibers

Following H-reflex recording, femoral nerve was cut in its proximal part to only record the afferent activity. The distal part of the femoral nerve was placed on bipolar reception electrode and immersed in paraffin oil. Electrical signal, recorded in absence of any movement, was amplified (10–100 K) with a differential amplifier (P2MP^®^ SARL, Marseille, France), filtered (pass-band filter: 30 Hz to 10 kHz) and discriminated in order to exclude aberrant signals (P2MP^®^ SARL). Discharge rate was calculated with a software (Biopac MP150^®^ and AcqKnowledge^®^ software, Biopac System, California, USA) and the variation of afferent discharge frequency during data acquisition was calculated off-line using an in-house MatLab^®^ program. First of all, basal discharge of afferent fibers (without chemical injection) was recorded during 230 seconds. This baseline was considered acceptable if discharge variation did not exceed 3%. Thus, discharge variations recorded following chemical injections were considered to an increase in III and IV afferent activity and not to variations due to environmental conditions. Afferent activity was recorded during 60 s after an injection of a mixture of KCl/AL (0.5 ml) with a concentration of 5/15 mmol/l or 10/25 mmol/l. These concentrations are close to those naturally released by rat hindlimb muscles during physical activity. To avoid chemical accumulation and to give chemicals time to be degraded, a delay of 10 min was left between the two injections^41^. Post-injection afferent discharge was compared to pre-injection activity and expressed as a percentage of baseline discharge frequency (%).

### Histological analysis

At the end of the electrophysiological recordings, animal were killed with an overdose of anesthetic (Pentobarbital sodium, 180 mg/kg, i.p., Exagon^®^, Axience, Pantin, France) and left knee joint of Control and MIA groups were removed from rat hindlimb. Femoral and tibial bones were sectioned at 0.5 cm on both side of the knee joint. Samples were completely cleaned of all surrounding soft tissues and fixed in 4% formaldehyde (Merk Millipore^®^, Fontenay sous Bois, France) in 0.01 mol/l phosphate buffer saline (Sigma-Aldrich^®^) at pH 7.4 for 10 days. Samples were rinsed in PBS (pH 7.4) and decalcified in 12% of ethylenediaminetetraacetic acid (EDTA, Sigma-Aldrich^®^) during 2 weeks. Radiographies were regularly performed to control decalcification. When decalcification was achieved, samples were washed in deionized water and dehydrated in alcohol baths of increasing concentration (60°, 80°, 95°, 100°). Samples were left immersed 2 days in each bath at room temperature and finally cleaned with tissue clearing agent (Histo-Clear-II^®^, National diagnostics^®^, Atlanta, USA). Knee joints were infiltrated with immersion in three successive baths of liquid paraffin during 4 hours and embedded in solid paraffin (polyisobutylene mixture, Paraplast plus^®^, Sigma-Aldrich^®^). Blocks of paraffin containing rat knee joints were cut in the sagittal plane using a microtome (Leica^®^ RM 2265, Wetzlar, Germany). Sections (thickness: 8 μm) were stained with toluidine blue (pH 5), dehydrated and mounted with rapid mounting medium for microscopy (Entellan^®^, Merk Millipore^®^). Toluidine blue was used to highlight metachromatic properties of the cartilage. This cationic metachromatic blue dye binds to the cartilage proteoglycans leading to a coloring of the matrix in purple-red^42,43^.

Histological sections were analyzed with an optical microscope (Olympus BX40, Olympus France SAS, Rungis, France) photographed using a CDD camera (Olympus DP21, Olympus France SAS) and evaluated using Mankin score for osteoarthritis. This score makes it possible to classify sections into no (scores: 1 to 5), moderate (scores: 6 to 10) and severe (scores: 11 to 14) osteoarthritis according to surface structure, chondrocytes aspect and coloration features^44^.

### Statistical analysis

All analyses were performed using statistical software (SigmaStat, Systat Software, Inc., San Jose, USA). Population normality was verified using the K² D’agostino-Pearson test. The results were processed through an analysis of variance (ANOVA). Results from DWB were analyzed by a three-way ANOVA (group effect, paw effect and time effect) while kinematics results were analyzed by a two-way ANOVA (group effect and time effect). Concerning kinematics analysis, ANOVA was carried out for each of the 30-time periods of walking. Finally, regarding electrophysiological data, a two-way ANOVA was carried out (group effect and time effect for the H-reflex; group effect and dose effect for afferent fiber activity). Data were expressed as mean ± SD. Results were considered significant if the p-value fell below 0.05.

## Results

### Behavioral tests

#### Dynamic weight-bearing

**Figure 2:**
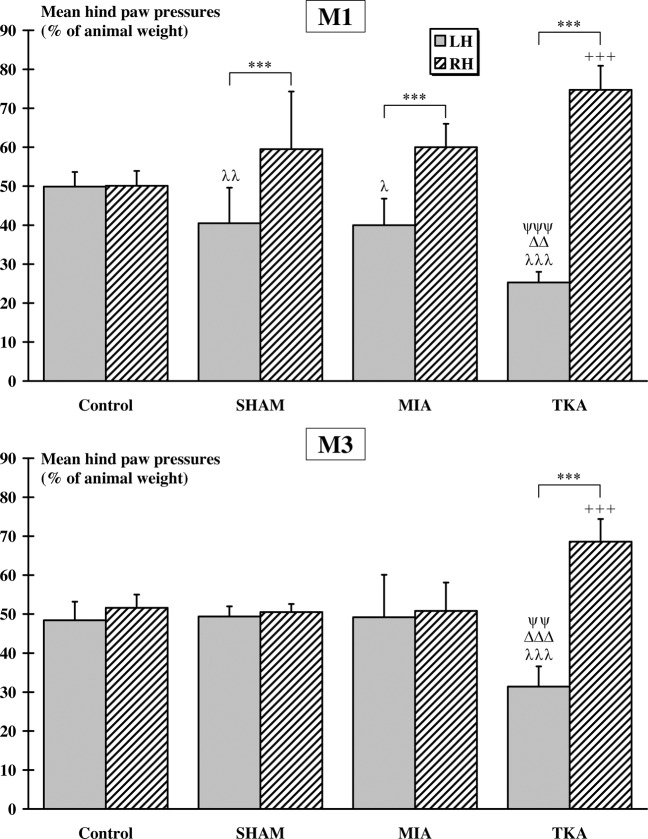
*Dynamic weight-bearing: rearing condition (two hindpaws)*. In each group, significant differences between the left (LH) and right (RH) hindpaws are indicated by *. Significant differences with the Control group are indicated by λ (for LH) and + (for RH). Finally, significant differences between the LH mean pressures of the TKA group and the LH mean pressures of the SHAM and MIA groups are indicated by Δ (*vs*. SHAM) and ψ (*vs*. MIA), respectively. (2 symbols: p < 0.01 and 3 symbols: p < 0.001).

Normalized pressures exerted by the two hindpaws are shown in Fig. 2. After one month (M1), no difference was observed between mean pressures exerted by left hindpaw (LH) and right hindpaw (RH) for Control group (LH = 49.9 ± 3.7%, RH = 50.1 ± 3.8%). However, mean RH pressures were significantly higher than mean LH pressures for SHAM (LH = 40.5 ± 9.1%, RH = 59.5 ± 14.8%, p < 0.001), MIA (LH = 40.0 ± 6.8%, RH = 60.0 ± 6.0%, p < 0.01) and TKA (LH = 25.3 ± 2.7%, RH = 74.7 ± 6.2%, p < 0.001) groups. Mean LH pressures exerted by Control group were significantly higher than the corresponding pressures of SHAM (p < 0.01), MIA (p < 0.05) and TKA (p < 0.001) groups. Furthermore, mean LH pressures of TKA group were lower than mean LH pressures recorded in the SHAM (p < 0.01) and MIA (p < 0.001) groups. Finally, mean RH pressures measured in the TKA group were significantly higher than mean RH pressures recorded in the Control group (p < 0.001). After 3 months (M3), no difference in mean LH and RH pressures was observed between the Control (LH = 48.4 ± 4.8%, RH = 51.6 ± 3.4%), SHAM (LH = 49.5 ± 2.6%, RH = 50.5 ± 2.1%) and MIA (LH = 49.2 ± 10.9%, RH = 50.8 ± 7.3%) groups. However, a significant higher (p < 0.001) mean RH pressures (RH = 68.6 ± 5.8%) was measured in the TKA group compared to the mean LH pressures (LH = 31.4 ± 5.1%). Furthermore, the mean RH pressures measured in the TKA group were significantly higher (p < 0.001) than mean RH pressures of the Control group. Finally, the mean LH pressures of the TKA group were significantly lower than the mean LH pressures recorded in the Control (p < 0.001), SHAM (p < 0.001) and MIA (p < 0.01) groups.

**Figure 3:**
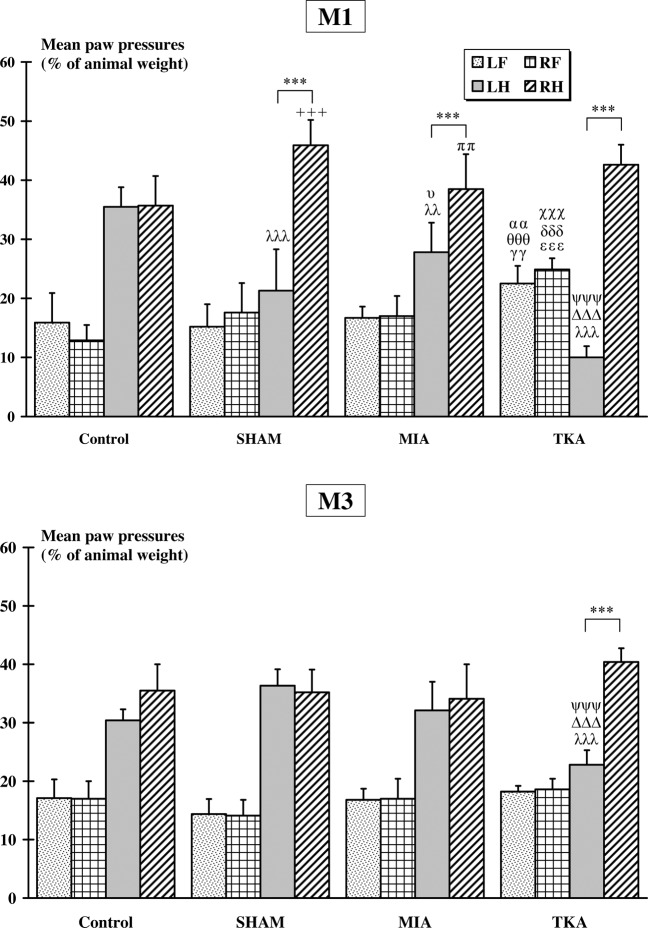
*Dynamic weight-bearing: normal condition (four paws)*. In each group, significant differences between the left (L) and right (R) fore (F) or hind (H) paws are indicated by *. For a given paw, significant differences with Control group are indicated by λ (for LH), + (for RH), γ (for LF) and ε (RF). Significant differences between the mean pressures of the TKA group and the mean pressures of the SHAM and MIA groups are indicated by Δ (LH, *vs*. SHAM), ψ (LH, *vs*. MIA), δ (RF, *vs*. SHAM), χ (RF, *vs*. MIA), θ (LF, *vs*. SHAM) and α (LF, *vs*. MIA). Finally, differences between SHAM and MIA groups are indicated by υ (for LH) and π (for RH). (1 symbol: p < 0.05; 2 symbols: p < 0.01 and 3 symbols: p < 0.001).

Normalized pressures exerted by the four paws are shown in Fig. 3. At M1, no significant difference was observed between mean pressures recorded in left (LF) and right (RF) forepaws for all groups (Control: LF = 15.9 ± 5.0%, RF = 12.9 ± 2.6%; SHAM: LF = 15.2 ± 3.8%, RF = 17.6 ± 5.0%; MIA: LF = 16.8 ± 1.9%, RF = 17.0 ± 3.4%; TKA: LF = 22.0 ± 3.0%; RF = 24.9 ± 1.9%). Furthermore, mean pressures recorded in the TKA group were significantly higher than those recorded in the Control (p < 0.01 for LF and p < 0.001 for RF), SHAM (p < 0.001 for both forepaws) and MIA (p < 0.01 for LF and p < 0.001 for RF) groups. Concerning the hindpaws mean pressures, no difference was observed between LH (35.5 ± 3.3%) and RH (35.7 ± 5.0%) for Control group. However, in all other groups, the mean LH (SHAM: 21.3 ± 7.0%; MIA: 27.8 ± 5.0%; TKA: 10.0 ± 1.9%) pressures were significantly (p < 0.001) lower than the mean RH pressures (SHAM: 45.9 ± 4.3%; MIA: 38.5 ± 5.9%; TKA: 42.6 ± 3.4%). The mean LH pressures were significantly higher in Control group compared to mean pressures recorded in SHAM (p < 0.001), MIA (p < 0.01) and TKA (p < 0.001); the latter revealing also significant (p < 0.001) lower mean pressures compared to all the SHAM and MIA groups. The mean LH pressures in the MIA group were significantly (p < 0.05) higher than that of the SHAM group. In the same way, the mean RH pressures in the SHAM group were significantly (p < 0.001) higher than that of the Control group. Finally, the mean RH pressures in the MIA group were significantly lower (p < 0.01) than that of the SHAM group. At M3, no significant difference was observed between LF (Control: 17.1 ± 3.2; SHAM: 14.4 ± 2.6; MIA: 16.8 ± 1.9; TKA: 18.2 ± 1.0) and RF (Control: 17.0 ± 3.0; SHAM: 14.1 ± 2.7; MIA: 17 ± 3.4; TKA: 18.6 ± 1.8) mean pressures for all groups. Furthermore, no significant difference was observed between LH and RH mean pressures in Control (LH = 30.4 ± 1.9%, RH = 35.5 ± 4.5%), SHAM (LH = 36.3 ± 2.8%, RH = 35.2 ± 3.9%) and MIA (LH = 32.1 ± 4.9%, RH = 34.1 ± 5.9%) groups. Only, TKA group presented a significant difference (p < 0.001) between the mean pressures measured in the hindpaws (LH = 22.8 ± 2.5%, RH = 40.4 ± 2.3%). Finally, mean LH pressures recorded in the TKA group were significantly lower (p < 0.001) than those of Control, SHAM and MIA groups.

#### Gait kinematic

**Figure 4:**
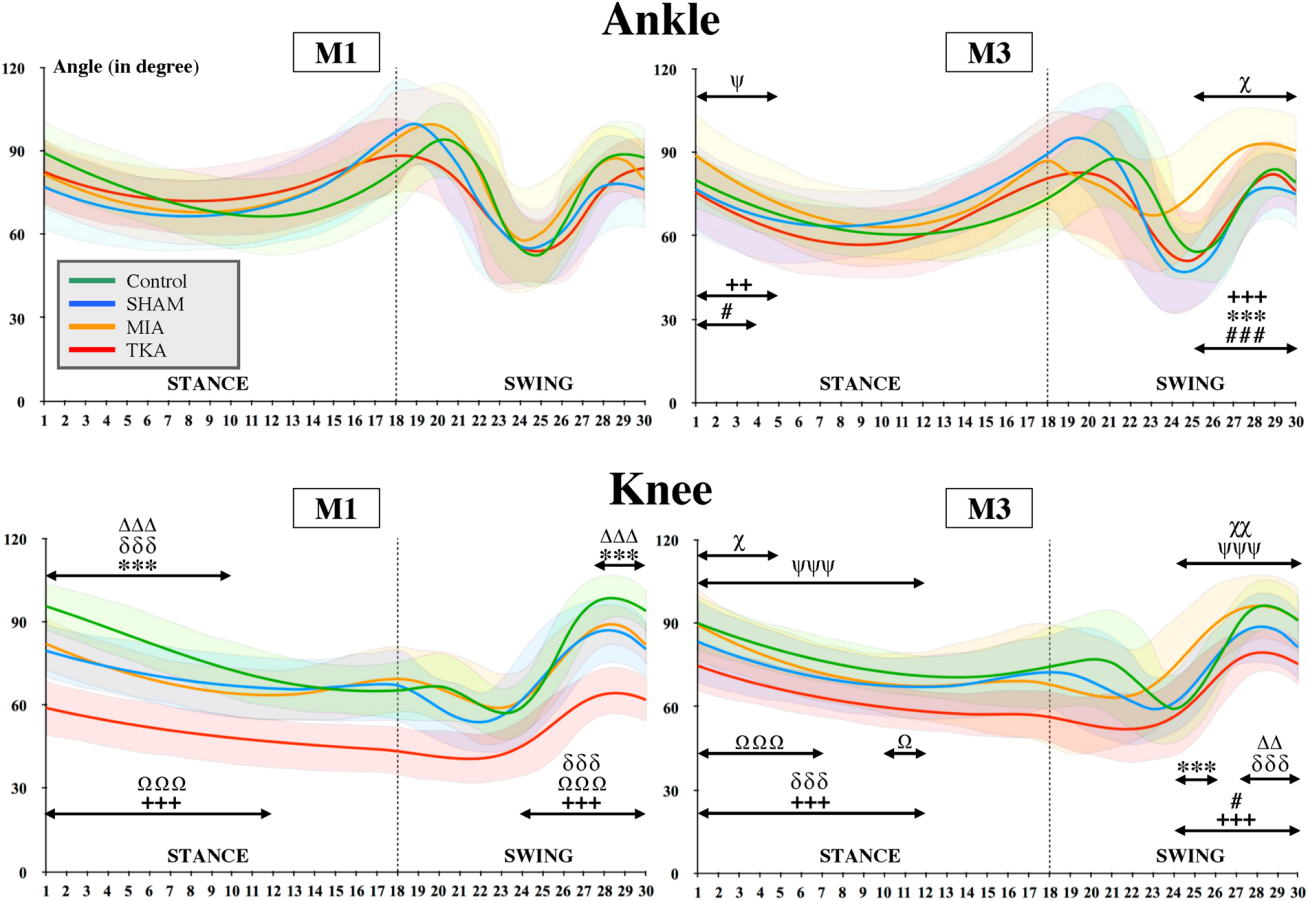
*Gait kinematics*. Representation of the evolution of the mean ankle and knee angles (curves) during a gait cycle (stance and swing phases). Standard deviations are represented by the cloud around the curves. Each group are identified by a color (green: Control, blue: SHAM, orange: MIA and red: TKA). Differences between groups and between M1 and M3 are indicated by the following symbols: # (MIA *vs*. SHAM), *(MIA *vs*. Control), +(MIA *vs*. TKA), Ω(TKA *vs*. SHAM), δ(TKA *vs*. Control), Δ(SHAM *vs*. Control), ψ(TKA at M1 *vs*. TKA at M3) and χ(MIA at M1 *vs*. MIA at M3). (1 symbol: p < 0.05; 2 symbols: p < 0,01 et 3 symbols: p < 0.001).

Evolution of ankle and knee angles during normalized gait cycles is illustrated in Fig. 4. At M1, no difference was observed between groups in ankle angles during the stance and the swing phase. However, at M3, ankle angles of the MIA group were significantly higher at the beginning of stance phase compared to the SHAM (p < 0.05) and TKA (p < 0.01) groups and at the end of the swing phase compared to all the other groups (p < 0.001 for all). In addition, at the end of the swing phase, the MIA group showed significant (p < 0.05) higher ankle angles at three months compared to those measured at one month. Finally, at the beginning of the stance phase, ankle angles of TKA group were significantly higher (p < 0.05) at M1 than at M3. Concerning knee angles, at M1, values of the Control group in the first half of the stance phase were significantly higher (p < 0.001) than values of the SHAM and MIA groups. Values of the Control group was also significantly higher than values of the SHAM (p < 0.001) and MIA (p < 0.01) groups at the end of the swing phase. In addition, TKA knee angles were significantly lower (p < 0.001) than all the other groups during the first 66% of stance phase and during the last half of swing phase. At M3, TKA knee angles were also lower than those of Control (p < 0.001), SHAM (p < 0.05) and MIA (p < 0.001) groups during the first 66% of stance phase. Furthermore, knee angles of the MIA group were significantly higher (p < 0.001) to those of Control group at the beginning of the second half of swing phase and higher for the entire second half part than those of SHAM (p < 0.05) and TKA (p < 0.001) groups. At the end of swing phase, knee angle values of the Control group were significantly higher than those of SHAM (p < 0.01) and TKA (p < 0.001) groups. In addition, knee angles of TKA group were significantly higher (p < 0.001) at M3 compared to M1 during the first 66% of stance phase and during the last half of swing phase. Finally, MIA group showed also higher knee angles during the first third of stance phase (p < 0.05) and the last half of swing phase (p < 0.01) at M3 than at M1.

### Electrophysiological recordings

#### H-reflex

**Figure 5:**
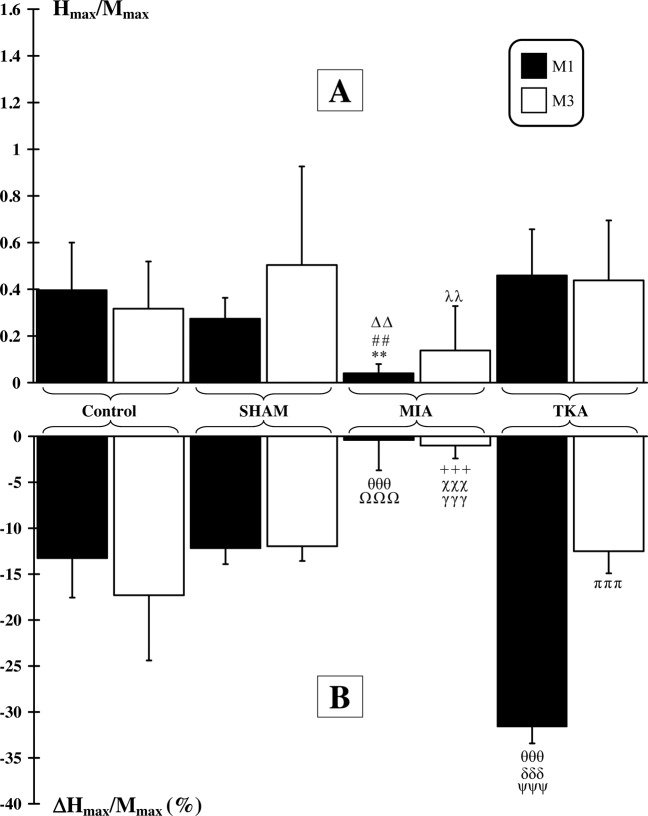
*H-reflex*. (**A**) H_max_/M_max_ ratio. At M1, significant differences with Control group are indicated by *. Significant differences between the SHAM and MIA groups are indicated by #. Significant differences between the MIA and TKA groups are indicated by Δ. At M3, significant differences between MIA and TKA group are indicated by λ. (**B**) H_max_/M_max_ ratio variation to injection of a mixture of KCl and LA (10–25-mM). At M1, significant differences with Control group are indicated by θ. Significant differences between the SHAM and TKA groups are indicated by δ. Significant differences between the MIA and TKA groups are indicated by ψ. Significant differences between the MIA and SHAM groups are indicated by Ω. At M3, significant differences with Control group are indicated by+. Significant differences between the MIA and TKA groups are indicated by χ. Significant differences between the MIA and SHAM groups are indicated by γ. Finally, in the same group, significant difference between M1 and M3 are indicated by π. (2 symbols: p < 0.01 and 3 symbols: p < 0.001).

H_max_/M_max_ ratio and its variation following KCl and AL injections are presented in Fig. 5. Before injection, at M1, basal value of H_max_/M_max_ ratio in the MIA group (0.04 ± 0.04) was significantly lower (p < 0.01) compared to values calculated in Control (0.39 ± 0.20), SHAM (0.27 ± 0.10) and TKA (0.46 ± 0.19) groups. At M3, no difference was observed between Control (0.32 ± 0.20) and other groups (SHAM: 0.50 ± 0.42; MIA: 0.14 ± 0.19; TKA: 0.44 ± 0.26). However, H_max_/M_max_ ratio was significantly lower (p < 0.01) in the MIA group compared to the TKA group. Finally, no difference was observed in each group between values calculated at M1 and M3. After chemical injection, at M1, no difference was observed between Control (−13.26 ± 4.3%) and SHAM (−12.17 ± 1.75%) groups. However, TKA group showed a significant (p < 0.001) higher H_max_/M_max_ variation (−31.62 ± 1.8%) than Control, SHAM and MIA (−0.4 ± 3.3%) groups. In addition, H_max_/M_max_ variation in the MIA group was also significantly lower (p < 0.001) than other groups. At M3, no difference was observed between Control (−17.30 ± 7.1%), SHAM (−11.96 ± 1.60%) and TKA (−12.50 ± 2.4%) groups. However, H_max/_M_max_ variation was significantly (p < 0.001) lower for MIA (−1.0 ± 1.4%) group compared to all other groups. Finally, except for TKA group that showed a significant (p < 0.001) lower H_max_/M_max_ value at M3 than at M1, no difference was observed between values calculated at M1 and M3 for the others groups.

#### III and IV afferent fibers

**Figure 6:**
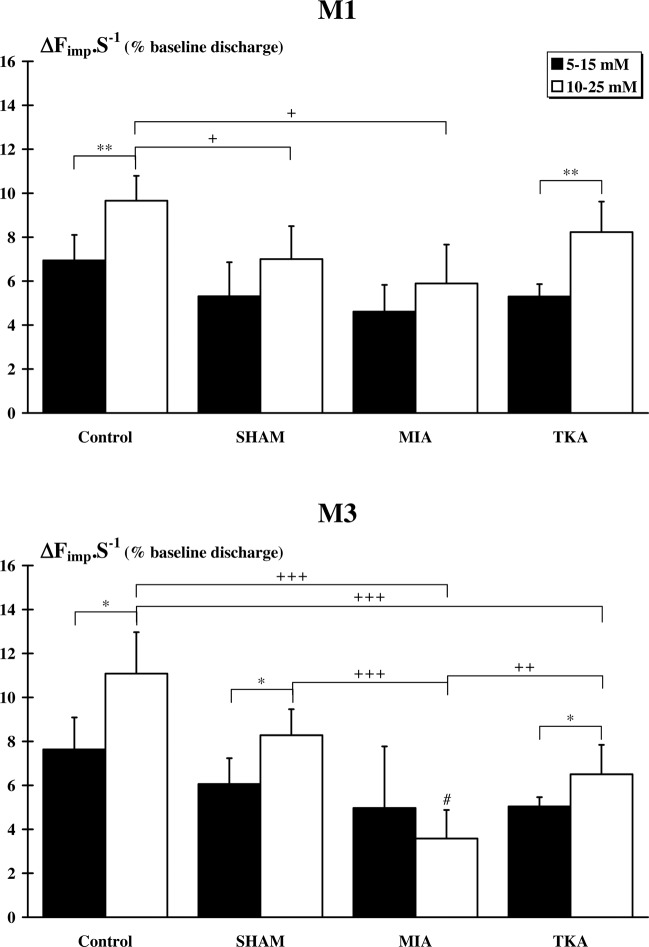
*Afferent responses after injections of a mixture of KCl and LA*. Discharge variation was expressed as a percentage of the baseline discharge. In each group, significant differences between the two mixtures (5–15 mM and 10–25 mM) are indicated by *. For a given dose, significant differences between group are indicated by + . Finally, in each group, significant differences between M1 and M3 for a given dose are indicated by #. (1 symbol: p < 0.05; 2 symbols: p < 0.01 and 3 symbols: p < 0.001).

Data of afferent discharge variation following chemical injections are presented in Fig. 6. At M1, all group responded to chemical injections. However, no difference was observed between all groups following KCl/AL 5–15 mM injection (Control: 6.94 ± 1.16%; SHAM: 5.31 ± 1.55%; MIA: 4.61 ± 1.22%; TKA: 5.30 ± 0.56%). After KCl/AL 10–25 mM injection, only Control (9.66 ± 1.13%) and TKA (8.23 ± 1.39%) groups showed higher (p < 0.01) afferent discharge variation compared to 5–15 mM injection. Furthermore, following 10–25 mM injection, Control afferent discharge variation was higher (p < 0.05) than variation for SHAM (7.01 ± 1.51%) and MIA (5.89 ± 1.77%) groups. At M3, all group responded to chemical injections. However, afferent discharge variation was not different between groups following KCl/AL 5–15 mM injections (Control: 7.63 ± 1.46%; SHAM: 6.06 ± 1.17%; MIA: 4.97 ± 2.8%; TKA: 5.04 ± 0.42%). After KCl/AL 10–25 mM injection, afferent discharge variation was significantly higher (p < 0.05) for Control (11.08 ± 1.89%), SHAM (8.28 ± 1.18%) and TKA (6.50 ± 1.34%) groups compared to 5–15 mM injection. However, no difference was observed concerning discharge variation in the MIA group between 10–25 mM (3.58 ± 1.30%) and 5–15 mM injections. Afferent discharge variation of MIA group following 10–25 mM injection was significantly lower than discharge of the other groups (Control and SHAM: p < 0.001; TKA: p < 0.01). In addition, Control group showed higher (p < 0.001) afferent discharge variation than TKA group. Finally, afferent discharge variation of MIA group was lower (p < 0.05) at M3 than at M1.

### Histological analysis

**Figure 7:**
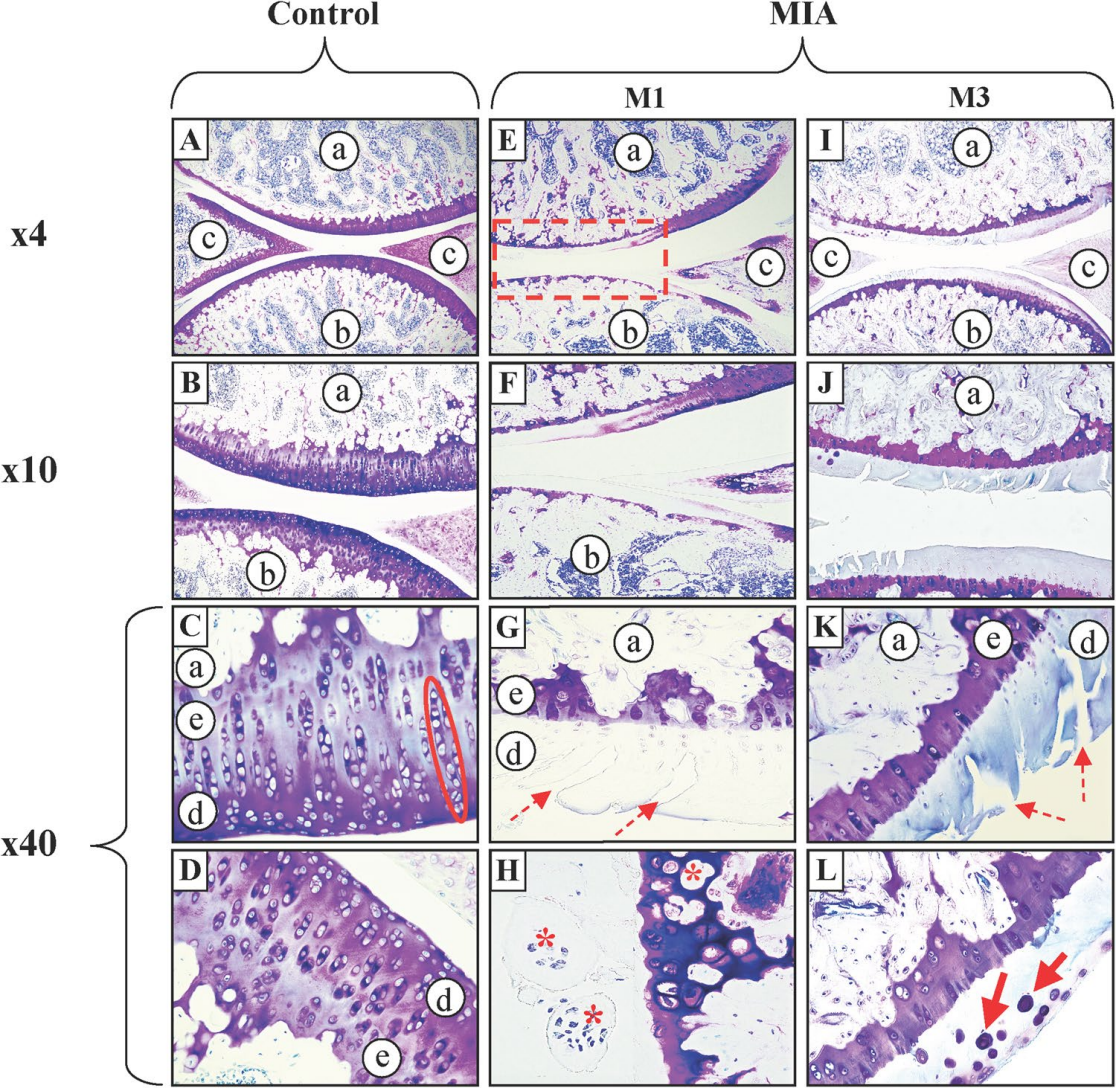
*Histological analysis*. Pictures of sagittal sections of knee joint from Control (A-D) and MIA groups at M1 (E-H) and M3 (I-L) stained with toluidine blue. Femur (**a**), Tibia (**b**), Menisci (**c**), Hyaline cartilage (**d**) and Calcified cartilage (**e**). In the Control group (**A-D**), the cartilage is healthy. Lower magnification (**A–B**) shows that joint surfaces are smooth, intact and contain a high concentration of proteoglycans (in purple-red). High magnification (**C–D**) reveals a linear organization of chondrocytes (encircled in red) which are dense and homogeneous throughout the cartilage and with a healthy appearance. At M1 (**E–H**), in the MIA group, the thickness of the cartilage is reduced as the number of proteoglycans in the hyaline cartilage of both femur and tibia (red rectangle). Calcified cartilage shows a reduced thickness, a low density of chondrocytes and cluster with residues of cells (*). Joint surfaces are cracked (dotted arrow) and cartilaginous remnants still containing a few cells are observed in the intra-joint space (*). At M3, proteoglycans are very rare from the whole joint surfaces. Joint surfaces present many deep cracks and a very marked hypocellularity with cells having a very condensed appearance (arrows).

Representative pictures of knee sections of Control and MIA groups at M1 and M3 are shown in Fig. 7. In the Control group, knee joints presented a thick layer of hyaline cartilage which demonstrated high content in proteoglycans and glycosaminoglycans. A high density of chondrocytes was also observed. These cells had a completely healthy appearance and were linearly organized. In addition, all the joint surfaces were smooth. From these observations a Mankin score of 0 was assigned to the Control group.

One month following intra-joint MIA injection, hyaline cartilage of MIA group showed important purple coloration deficits which highlighted a major decrease of proteoglycans and glycosaminoglycans, particularly at the center part of the joint where biomechanical constraints are important. Density of chondrocyte was poor and cells were disorganized. In addition, deep cracks were observed at joint surfaces and cartilaginous remnants were present in the intra-joint space. From these observations a Mankin score of 11 was assigned to the MIA group at M1.

Three months following intra-joint MIA injection, there was poor hyaline cartilage staining which extended over all joint surfaces. Calcified cartilage did not seem to be damaged and still had a healthy appearance. Nevertheless, joint surfaces presented many deep cracks and a very low cell density. Some chondrocytes had a condensed appearance which seemed to reflect an apoptotic process. From these observations a Mankin score of 12 was assigned to the MIA group at M3.

## Discussion

Nowadays, total knee arthroplasty (TKA) procedure is steadily increasing in most industrialized countries. This dramatic trend expected to continue in the coming years due to population aging, increasing physical activity and obesity progression^8,45^. Generally, resorting to total knee prosthesis establishment occurs for people suffering from severe knee osteoarthritis (OA), with severe pain and/or significant loss of autonomy due to the resulting motor limitations. TKA involves a heavy surgery engendering major motor deficits, mainly characterized by muscular atrophy of stabilizing knee muscles (quadriceps and hamstring), strength loss, voluntary activation deficits, and proprioceptive/kinesthetic deficits^12,16,46,47^. Paradoxically, even though motor deficits are necessarily due to motor control adjustment processes, very few studies have investigated these processes following a TKA altering a large number of sensory receptors and consequently disturbing the functioning of the sensorimotor loop^48^. Thus, any change in the sensorimotor loop may be at the origin of motor deficits and their persistence in time.

In the present study, for the first time, we demonstrate that knee OA altered considerably the responses of III and IV afferent fibers to their known stimuli^49^ and consequently their ability to modulate the sensorimotor loop, although, paradoxically, motor deficits seemed relatively light suggesting compensatory mechanisms. On the contrary, we demonstrate that TKA slightly altered the afferent responses and the sensorimotor loop but that motor deficits were more severe suggesting a central adaptive process.

In accordance with several previous studies^50–53^, we observed that MIA injection induced severe cartilage damage demonstrating that this method was effective to induce severe OA. Thus, as in human clinical cases, our rat model is suitable for total knee arthroplasty.

In our study, we noted, as for patients with OA, that joint cartilage impairment did not necessarily lead to significant functional limitations on the short term^54,55^. Moreover, it was reported that degenerative OA processes are not linear^56^. Indeed, the phases of stabilization and aggravation that follow one another could potentially explain the largest deficits observed in the first months (M1) compared to those in the third month (M3) even if at M3 the cartilage was more degraded. Moreover, habituation to pain could explain the weight bearing distribution on two and four paws in MIA group.

Regarding gait kinematics, although knee angular variations in the MIA group were close to those of Control group, ankle angle values suggested that rats with OA adopt a strategy in which the ankle participated differently to locomotion in order to compensate knee joint deficits. This strategy had already been observed in people with knee OA^2,3^. Indeed, the literature reports that people with knee arthritis readjust their motor control to reduce knee contribution and increase that of ankle^57^. Although this study assessed knee OA effects on a period of three months, degenerative features of OA suggests that the relatively “good” functionality observed in MIA groups could be temporary. Indeed, like for patients with OA, we can expect that, with time, the progressive degradation of joint cartilage and pain will significantly alter knee functionality. The choice to implant our prosthesis model is then quite justified.

Weight distribution (DWB) indicated changes from M1 to M3 in the mean pressures of the forepaws in the TKA group. Indeed, at M1, the pressures exerted by the forepaws in the TKA group were significantly higher than those of the other groups indicating a posterior-anterior compensation in which the forepaws were more solicited to lighten the weight in the posterior part of the body. At M3, a rebalancing was observed with an increase in the mean pressures exerted by the operated leg that may indicate a recovery. However, this recovery was incomplete because mean pressures on the operated side remained lower than those of the Control and SHAM groups and because an imbalance persists between the two anterior legs.

Gait kinematics revealed that animals in which a total knee prosthesis was implanted were able to walk but were slightly leaning on the operated limb. Furthermore, results suggested that these implanted animals presented some recoveries characterized by a reduction of functional deficits with time, i.e., the deficits were lower at M3 compared to those at M1. These results were particularly encouraging because they concretely validated our prosthesis design anatomically and biomechanically adapted to rat knee.

More precisely, at M1, knee angles of TKA group were significantly lower than those of the three other groups at the beginning of stance phase (at the beginning of the forward weight transfer) and at the end of swing phase (when hindpaw extends to prepare the ground contact). These results highlight a strong alteration of knee extension capacity for implanted animals. This is in accordance with several human clinical studies demonstrating major weakness of quadriceps, which is the main knee extensor muscle group^12,16,46,47,58,59^. At M1, knee angles of TKA group were still different compared to Control, SHAM and MIA groups. However, there was a marked improvement in gait kinematics, both at the beginning of the stance phase and at the end of swing phase compared to knee angles at M1.

Unlike the MIA group, there did not seem to be any ankle compensation strategy that could be explained by the congruence of tibial and femoral parts of the prosthesis ensuring a low level of joint constraint and allowing to implanted animals to adopt the same walking strategy that Control group. Gait of TKA group remained deficient but recovered tending towards normality and not towards a phenomenon of compensation as described previously for the animals with knee OA. In the MIA group, joint surface incongruence involved a change in motor strategy with changes of ankle angles during locomotion.

In the MIA group, the basal value of the H_max_/M_max_ ratio and changes of this ratio following activation of the III and IV afferent fibers were very low at M1 and M3. On the contrary, H_max_/M_max_ ratio was decreased in all other groups confirming previously results indicating that specific activation of III and IV by KCl and LA was followed by a decrease in H_max_ amplitude^40^. Our results may suggest that knee OA disturbed the afferent fibers to modulate the motor order. This hypothesis was reinforced by the fact that, following KCl/LA 10–25 mM injection, increase in discharge frequency of III and IV afferent fibers was lower in MIA group compared to the other groups. Our results were consistent with previous electrophysiological studies demonstrating a direct relationship between OA severity and afferents sensitivity^60,61^. Moreover, many studies have suggested that joint damage can lead to arthrogenic muscle inhibition^62–65^ that is a reduction in spinal reflexes excitability, including H-reflex by sensory information originating from damaged joint areas^62,64^. Thus, we can’t not excluded that knee joint damages could inhibit motor units excitability of quadriceps. Moreover, inflammatory reaction associated with joint surfaces degradation is likely to contribute to the severity of nervous deficits. Indeed, cartilage damage promotes the release of inflammatory mediators such as cytokines including interleukins (IL-1β and IL-6) and tumor necrosis factors (TNF-α), and degradation enzymes such as metalloproteinases (MMPs)^3,66,67^. Some of these substances are known to stimulate III and IV afferent fibers^24,68,69^. Thus, a down-regulation or an over-activation of III and IV afferent fibers could explain the lack of response following high dose of KCl/LA injection and then a decreased sensory signal to different integrative centers. Finally, our results indicate that knee OA (MIA group) did not greatly affect motor activity suggesting the existence of compensatory mechanisms related to nervous system adaptation in response to changes in activities from III and IV afferent fibers.

In the TKA group, the basal value of the H_max_/M_max_ ratio was not different from Control group either at M1 or at M3. However, at M1, the H_max_/M_max_ ratio variation following III and IV afferent fibers activation was greater than those other groups that could be explained by changes induced by arthroplasty surgery. Indeed, cruciate ligaments section, menisci removal and joint surfaces shortening may suppress inflammatory components, partially alter joint afferents and then arthrogenic muscle inhibition^63,64,70^. At M3, the H_max_/M_max_ ratio variation following III and IV afferent fibers activation seemed to normalize; i.e., the variation was similar to that of the Control and SHAM groups. These results could be explained by an adaptive strategy to the remaining information from joint afferents.

As a methodological limitation, it could be argued that our results are difficult to transpose to human model. However, our model of chemically induced osteoarthritis was demonstrated to be closer to clinical situation^39^. Another lock consists in the complexity of the human knee prosthesis since this prosthesis is composed with a femoral part and a tibial part with two components. For the latter, a metallic part is directly inserted into the tibial plateau and an upper part is made in ultra-high molecular weight polyethylene (UHMWPE). Consequently, this piece allows to lower friction and therefore decreases the delamination risk between the two metallic parts of the prosthesis. However, to simplify the problem in our model, we choose to work on a prosthesis made with only two parts. The tibial part was made in PEEK which is known to provide good osseointegration without cytotoxicity and to provide a good friction coefficient with the metallic part. The femoral part was classically made in TiAl6V4.

In conclusion, the aim of the present study was to determine the origin of functional deficits after knee OA and TKA. Our results indicated that after 3 months, knee OA did not induce significant motor functional limitations. However, the damaged joint surfaces were associated to an alteration of the III and IV afferent responses and of changes in H_max_/M_max_ ratio following their stimulation. Furthermore, TKA was associated to greater motor limitations which were not associated to altered responses of III and IV afferents. Finally, paradoxically, the adjustment of the central drive by III and IV afferent fibers seemed again possible when the knee prosthesis was implanted that could suggest recovery. We conclude that neural changes observed when a total knee replacement was performed may disrupt or delay the locomotor recovery.

Because human clinical studies seem to report that functional recovery was not completed following TKA, it could be interesting to explore, in futures studies, the effect of improving the placement of the prosthesis and associated surgery procedures on sensorimotor loop changes. Finally, we are therefore on the initiative of an innovative “research and development” platform aimed at optimizing the clinical management of patients using TKA thanks to research carried out on an animal model: the rat. The innovation of a prosthesis adapted to this model allows us to explore in a more invasive way consequences of arthroplasty on neuromuscular adaptation. It will therefore be possible to work upstream to optimize the functional recovery of patients but also to find a consensus as to the best management strategy to adopt. Furthermore, our prosthesis model could allow *in vivo* testing of the effectiveness of innovative materials that do not yet have an orthopedic application.

## Data Availability

All data analyzed during this study are included in this publication. The datasets during and/or analyzed during the current study are available from the corresponding author on reasonable request.
